# The Whereabouts of an Ancient Wanderer: Global Phylogeography of the Solitary Ascidian *Styela plicata*

**DOI:** 10.1371/journal.pone.0025495

**Published:** 2011-09-23

**Authors:** Mari Carmen Pineda, Susanna López-Legentil, Xavier Turon

**Affiliations:** 1 Department of Animal Biology (Invertebrates), University of Barcelona, Barcelona, Spain; 2 Centre d'Estudis Avançats de Blanes, Consejo Superior de Investigaciones Científicas (CEAB-CSIC), Blanes, Spain; Barnard College, Columbia University, United States of America

## Abstract

Genetic tools have greatly aided in tracing the sources and colonization history of introduced species. However, recurrent introductions and repeated shuffling of populations may have blurred some of the genetic signals left by ancient introductions. *Styela plicata* is a solitary ascidian distributed worldwide. Although its origin remains unclear, this species is believed to have spread worldwide by travelling on ship's hulls. The goals of this study were to infer the genetic structure and global phylogeography of *S. plicata* and to look for present-day and historical genetic patterns. Two genetic markers were used: a fragment of the mitochondrial gene Cytochrome Oxidase subunit I (*COI*) and a fragment of the nuclear gene Adenine Nucleotide Transporter/ADP-ATP Translocase (*ANT*). A total of 368 individuals for *COI* and 315 for *ANT* were sequenced from 17 locations worldwide. The levels of gene diversity were moderate for *COI* to high for *ANT*. The Mediterranean populations showed the least diversity and allelic richness for both markers, while the Indian, Atlantic and Pacific Oceans had the highest gene and nucleotide diversities. Network and phylogenetic analyses with *COI* and *ANT* revealed two groups of alleles separated by 15 and 4 mutational steps, respectively. The existence of different lineages suggested an ancient population split. However, the geographic distributions of these groups did not show any consistent pattern, indicating different phylogeographic histories for each gene. Genetic divergence was significant for many population-pairs irrespective of the geographic distance among them. Stochastic introduction events are reflected in the uneven distribution of *COI* and *ANT* allele frequencies and groups among many populations. Our results confirmed that *S. plicata* has been present in all studied oceans for a long time, and that recurrent colonization events and occasional shuffling among populations have determined the actual genetic structure of this species.

## Introduction

Biological introductions have notably increased during the last century, posing a major threat to global biodiversity and altering the structure and function of many communities [1]–[7]. Despite some relatively recent attempts to buffer the ecological impact of these introductions [e.g. 8]–[10], oceans remain one of the most affected ecosystems [7], [11]–[17]. Among other transport vectors, non-native species arrive to new locations through ships' hulls and sea chests, in ballast water or with spats for mariculture. Thus, the increasing activity in maritime traffic and aquaculture has favoured the introduction of marine species all over the world [13], [18]–[19]. The establishment of new genetic variants and spread of exotic species has also been facilitated by a proliferation of harbours and other artificial structures along the coast [20]–[25].

Genetic diversity plays a crucial role on the successful establishment of an introduced species or variant in a new area [26]–[30]. The development of genetic tools and markers has widely contributed to enhance our knowledge on these species. A throughout assessment of the genetic structure of an introduced species, including its history of subdivision and gene flow, allows the identification of range expansions, colonization events, and an understanding of the invasive potential and the relative contributions of artificial and natural dispersal [e.g. 31]–[34].

The increasing pace of introductions has also fostered increased awareness. Monitoring and control programs have been established, and recent introductions are more easily detected and inventoried than in the past [e.g. 17]. However, historical invasions may still remain hidden. Some species could have arrived to a new location long before the distribution ranges of autochthonous species were assessed, and be now regarded as native [35], [36]. Cosmopolitan or broadly distributed species, particularly those thriving in harbours and artificial substrata, are likely to be “pseudoindigenous” species [36]. Lack of historical records in many regions, taxonomic flaws and cryptic speciation further complicate the issue [e.g. 37], [38]. In addition, and despite the new methods available [e.g., 33], our ability to extract information may be limited by our knowledge and access to native populations, recurrent introduction events, and shuffling of populations during a long period of time (i.e. centuries).

The paramount importance of ascidians for the study of marine introductions is well recognized, as they represent one of the most common invaders [39], [40]. Ascidians have short-lived larvae, thus anthropogenic transport can greatly increase their dispersal abilities. The rate of introduction of non-indigenous ascidians has been increasing in the last decades [40], mostly linked to ship traffic or aquaculture activities [e.g. 39], [41]–[45]. However, some species may have been translocated centuries ago and have now become ancient introductions whose origins are poorly known [46]. These ancient colonizers are often species commonly found in harbours and man-made substrates, have broad distribution ranges and, while naturalized in many areas, continue to be introduced in new regions of the globe [e.g. 47]–[50].

*Styela plicata* (Lesueur, 1823) (Tunicata, Ascidiacea) is a solitary ascidian commonly found inhabiting marinas and harbours of warm and temperate oceans, usually at high-densities. In spite of its broad geographical distribution, the native range of this species is not yet elucidated [46]. Evidence to date suggests that *S. plicata* is native to the NW Pacific Ocean [36], [51]–[54]. In fact, the description of this species was based on an individual found on a ship's hull in Philadelphia (NE USA), and no other individual was observed in the surrounding natural substrata [55]. All records of *S. plicata* are based on observations of man-made structures, except in Japan, where this species has been observed to grow in natural habitats [Nishikawa *pers. comm.*, 54]. A series of unique characteristics has allowed *S. plicata* to thrive in these diverse environments and outcompete other benthic invertebrates. *S. plicata* can physiologically adapt to widely fluctuating environments, particularly to changes in temperature and salinity [56], [57]. This species can also tolerate highly polluted waters [58], grows rapidly until reaching sexual maturity [59]–[61], and is capable of self-fertilization (authors' current research).

To gain insight into the invasive potential of this species, we analyzed the genetic structure of seventeen populations covering most of *S. plicata*'s distribution range. Using a mitochondrial (*COI*) and a nuclear (*ANT*) marker, we attempted to infer the global phylogeography of *S. plicata*, understand its dispersion patterns, and assess the diversity and connectivity of introduced populations.

## Methods

### Sampling

Samples of *Styela plicata* were collected in 2009 and 2010 from seventeen localities (Table 1): two from the Mediterranean Sea (Iberian Peninsula), three from the north-eastern Atlantic Ocean (Iberian Peninsula, Canary Islands), two from the north-western Atlantic Ocean (US east coast), one from the south-western Atlantic ocean (Brazil), five from the north-western Pacific Ocean (Japan and China), one from the south-western Pacific Ocean (Australia), one from the north-eastern Pacific Ocean (US west coast), and two from the south-western Indian Ocean (South Africa). These locations were chosen to cover as much of the distribution range of this widespread species as possible. All specimens were collected from artificial substrata (harbours, marinas or decks), except for one population collected from natural substratum in Sakushima Island (Japan). The shortest distance by sea between location pairs was calculated using the “measure line” tool of Google Earth (version 3.0, Google Inc., Amphitheatre Parkway, CA, USA). *S. Plicata* samples were obtained according to current Spanish regulations. Samples from outside Spain were collected by national researchers following their country regulations. This species is not protected by any law and all sampling was conducted outside protected areas.

**Table 1:** Population code, name, geographical region (including country), and GPS position for the populations of *Styela plicata* analyzed in this study.

Code	Population	Geographical Region/Country	Latitude/Longitude
AR	Arenys de Mar	NW Mediterranean Sea/Spain	41°34′36″N/2°33′32″E
JA	Javea	NW Mediterranean Sea/Spain	38°47′52″N/0°11′06″E
SP	San Fernando	NE Atlantic Ocean/Spain	36°27′36″N/6°12′13″W
FE	Ferrol	NE Atlantic Ocean/Spain	43°29′00″N/8°14′00″W
TEN	Tenerife	NE Atlantic Ocean/Spain	28°00′24″N/16°39′38″W
KNY	Knysna	SW Indian Ocean/South Africa	34°2′28″S/23°2′38″E
PE	Port Elizabeth	SW Indian Ocean/South Africa	33°57′49″S/25°38′16″E
NC	North Carolina	NW Atlantic Ocean/USA	34°8′24″N/77°51′44″W
SC	South Carolina	NW Atlantic Ocean/USA	32°12′57″N/80°46′49″W
CAL	California	NE Pacific Ocean/USA	32°47′00″N/117°09′00″W
BRA	Santa Catarina	SW Atlantic Ocean/Brasil	26°46′30″S/48°36′34″W
AM	Manly	SW Pacific Ocean/Australia	33°47′43″S/151°17′38″E
WAK	Wakayama	NW Pacific Ocean/Japan	34°11′17″N/135° 8′48″E
OKI	Okinawajima	NW Pacific Ocean/Japan	26°19′29″N/127°50′15″E
MIS	Misaki	NW Pacific Ocean/Japan	36°9′21″N/133°18′52″E
SKS	Sakushima Island	NW Pacific Ocean/Japan	34°43′00″N/137°02′00″E
HK	Hong Kong	NW Pacific Ocean/China	22°24′00″N/114°21′00′E

All specimens were collected from depths that ranged between 0 and 2 m by pulling up harbour ropes, removing specimens from submersed docks and pilings, or pulling individuals from rocky assemblages (natural population). Samples were dissected *in situ* and a piece of muscular tissue from the mantle or the siphon was immediately preserved in absolute ethanol. Ethanol was changed after a few hours, and samples were then stored at −20°C until DNA extraction.

### DNA extraction and sequencing

Total DNA was extracted using the REDExtract-N-Amp Tissue PCR Kit (Sigma-Aldrich). The universal primers LCO1490 and HCO2198 described in Folmer et al. [62] were used to amplify a fragment of the mitochondrial gene Cytochrome Oxidase subunit I (*COI*) from 368 individuals. The primer set designed by Jarman et al. [63] was used to amplify a fragment of the single-copy nuclear Adenine Nucleotide Transporter (*ANT*) gene. Based on the resulting sequences, we also designed the specific primers ANTf_Splic (5′-TTG GCA GCT GAT ATT GGA AAA GG-3′) and ANTr_Splic (5′-CCA GAC TGC ATC ATC ATK CG-3′), using the software Primer 3 v.0.4.0. [64]. Amplifications were carried out for 315 individuals using Jarman et al. [63] primers or the newly designed ones.

For both genes, amplifications were performed in a final volume of 20 µL using 10 µL of REDExtract-N-amp PCR reaction mix (Sigma-Aldrich), 1 µL of each primer (10 µM) for *ANT* or 0.8 µL for *COI*, and 2 µL of template DNA. The PCR program for *ANT* consisted of an initial denaturing step at 94°C for 2 min, 30 amplification cycles (denaturing at 94°C for 1 min, annealing at 58°C for 30 seconds and extension at 72°C for 30 seconds), and a final extension at 72°C for 6 min, on a PCR System 9700 (Applied Biosystems). The PCR program for *COI* was as described above, except for the amplification cycles, which were done at 94°C for 45 seconds, 50°C for 45 seconds and 72°C for 50 seconds. PCR products were purified using MultiScreen® filter plates (Millipore), labelled using BigDye® Terminator v.3.1 (Applied Biosystems) and sequenced on an ABI 3730 Genetic Analyzer (Applied Biosystems) at the Scientific and Technical Services of the University of Barcelona (Spain). Other samples were directly sent for purification and sequencing to Macrogen Inc. (Seoul, Korea Korea). From the resulting sequences, we discarded low quality reads for *ANT*, hence the lower number of specimens sequenced for this marker.

Sequences were edited and aligned using BioEdit® v.7.0.5.3 [65]. Some *ANT* sequences showed a deletion of 22 amino acids, thus heterozygotes had unequal lengths and had to be manually reconstructed by carefully analyzing both forward and reverse chromatograms. The allelic phase for *ANT* genotypic data was analyzed using fastPHASE 1.1 [66] implemented in the software DnaSP v.5 [67]. We also used the Recombination Detection Program (RDP3) [68] to test for recombination in our nuclear sequences. Sequences obtained in this study have been deposited in GenBank (accession numbers HQ916425 to HQ916446 for *COI*, and HQ916363 to HQ916423 for *ANT*).

### Population genetics

Number of alleles (*Nh*), gene diversity (*Hd*), and nucleotide diversity (*π*) were computed with DnaSP v.5 [67]. Allelic richness was calculated using the program Contrib v.1.02, which implements a rarefaction method to obtain estimates independently of sample size [106]. Genetix v.4.05.2 [69] was used to calculate inbreeding coefficients for the *ANT* data obtained with fastPHASE. The nearly unbiased estimation of allelic differentiation between populations was based on the adjusted D_est_ measure described by Jost [70], and calculated for each marker with SPADE [71]. The mean and SE values obtained with SPADE from 1,000 bootstrap replicates were used to calculate the confidence intervals and the degree of significance of the differentiation values (using a normal approximation). To correct for multiple comparisons, we set the p-value at 0.009, following the Benjamini and Yekutieli False Discovery Rate correction [72]. A value of D was deemed significant when the confidence interval around its mean did not contain 0. An analysis of molecular variance (AMOVA) was performed to examine population structure, and its significance was tested running 10,000 permutations in Arlequin v.3.1 [73]. The correlation of genetic and geographical distances was tested for all pairs of populations with a Mantel test [74] and 10,000 permutations using Arlequin.

Visual assessment of between-population differentiation was achieved by performing a discriminant analysis of principal components (DAPC) [75] on a dataset comprising information obtained from both genes. This recently developed technique extracts information from genetic datasets (multivariate in nature) by first performing a principal component analysis (PCA) on groups or populations, and then using the PCA factors as variables for a discriminant analysis (DA). The previous PCA step ensures that the variables input to DA meet the requirements of having less variables (alleles) than number of observations (individuals) and not having any correlation between variables [75]. DA seeks to maximize the inter-group component of variation. We performed DAPC analyses on both genes combined by using the adegenet package for R [76]. DAPC was performed (function dapc) using pre-defined groups corresponding to populations or groups of populations (see Results). Variables were centred but not scaled. In all analyses, 50 principal components of PCA were retained and input to DA. DA also provided estimates of the probability with which the analysis recovers the true membership of the individuals. Finally, in order to detect population growth and infer population demographic events, we computed Tajima's *D* [77], Fu's *F_s_* [78], *R_2_* [79], and the raggedness index (based on the mismatch distribution) [80], using DnaSP.

### Phylogenetic and phylogeographical analyses

The complete dataset was used to construct a median-joining network for each marker using Network v.4.5.1.6 [81]. Resulting loops for the *ANT* network were solved using criteria derived from the coalescent theory [82], [83]. For the *COI* network, only one loop was observed but it could not be resolved.

Phylogenetic analyses were conducted using *Styela gibbsii* as an outgroup (acc. number HQ916447 for *COI* and HQ916424 for *ANT*). The best-fit model of nucleotide substitution for each marker was selected using jModeltest v.0.1.1 [84], [85], with the Akaike Information Criterion (AIC) for *COI*, and the corrected version for small samples (AICc) for *ANT*. The positions corresponding to the indel detected for *ANT* were not included in the analysis (see Results). For Bayesian inference (BI), MrBayes v.3.1.2 software [86] was used to infer tree topologies, implementing the corresponding likelihood model for each gene fragment. For each gene, the program was run with 1 million generations with a sample frequency of 100 (10,000 final trees). After verifying that stationarity had been reached (i.e. the average standard deviation of split frequencies between two independent chains reached less than 0.01), the first 1,000 trees were discarded in both cases as burnin. Majority-rule consensus trees were generated from the remaining 9,000 trees. Bayesian posterior probabilities were used as a measure of support for the branch nodes obtained. The obtained trees were drawn with FigTree v.1.2.2. DnaSP was used to perform the McDonald & Kreitman test [87], and check whether patterns of variation among groups of sequences were consistent with predictions for a neutral model.

## Results

### Mitochondrial gene

**Figure 1:**
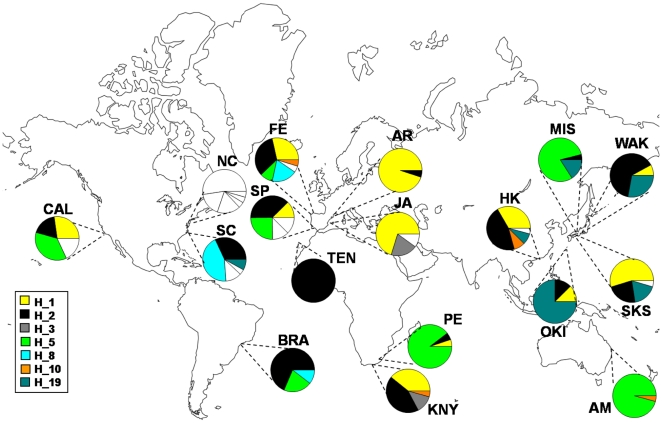
Map showing the sampling sites of *Styela plicata*. Pie charts represent haplotype frequencies for the *COI* gene in each population analyzed. Private haplotypes are shown in white.

For the mitochondrial *COI* gene, 368 sequences with a final alignment length of 624 bp were obtained. In total, we found 22 haplotypes with 38 polymorphic sites (6%), 6 of which corresponded to non-synonymous substitutions. The majority of haplotypes obtained (68%) corresponded to private haplotypes, most of which were found in the north-western Atlantic Ocean (Fig. 1). Remarkably, the six haplotypes found for the North Carolina population (NC) were private. The number of haplotypes per location ranged between one in Tenerife and six in Ferrol and North Carolina (Table 2, Table S1). Regarding the oceanic basins, the Atlantic and Pacific Ocean had higher haplotype diversity (17 and 8 haplotypes, respectively) than the Mediterranean Sea and the Indian Ocean (4 and 5 haplotypes, respectively; Table 2). Mean and total haplotype diversity (*Hd*) were 0.497 (±0.266 SD) and 0.810 (±0.010 SD), respectively. Mean nucleotide diversity was 0.0055 (±0.005 SD), while total nucleotide diversity (*π*) was 0.0135 (±0.0006 SD). Variation in haplotype and nucleotide diversity between populations within basins was considerable. For instance, the populations of Knysna (KNY) and Port Elizabeth (PE) located in the Indian Ocean, had a haplotype diversity of 0.668 and 0.205 respectively. The California population (CAL) presented the highest haplotype and nucleotide diversity values (0.800 and 0.01684, respectively; Table 2). The higher allelic richness values (obtained after rarefaction to a common sample size of 11 and 40 genes per populations and basins) were found for the San Fernando (SP, 3.747) and Ferrol populations (FE, 3.793), while the lower values corresponded to the populations of Manly (AM, 0.458) and Arenys de Mar (AR, 0.555). When comparing between basins, the Atlantic Ocean showed the highest allelic richness, whereas the Mediterranean Sea had the lowest value (Table 2).

**Table 2:** Diversity measures for the studied populations of *Styela plicata*.

Population	*COI*							*ANT*									
	*N*	*r*	*Hd*±SD		*π*±SD		*Nh* (private)	*N*	*r*	*Hd*±SD		*π*±SD		Nh (private)	*F_is_*	*H_exp_*	*H_obs_*
AR	20	0.555	0.100	(±0.088)	0.00016	(±0.00014)	2	19	3.733	0.620	(±0.072)	0.02012	(±0.00260)	6	0.241*	0.620	0.474
JA	20	1.785	0.484	(±0.113)	0.00388	(±0.00095)	3 (1)	20	3.307	0.494	(±0.088)	0.01670	(±0.00319)	5 (1)	0.802*	0.494	0.100
SP	16	3.747	0.775	(±0.068)	0.01484	(±0.00200)	5 (2)	17	8.434	0.791	(±0.065)	0.02831	(±0.00345)	11 (3)	0.266*	0.795	0.588
FE	21	3.793	0.795	(±0.051)	0.00835	(±0.00274)	6 (1)	13	7.363	0.822	(±0.059)	0.02258	(±0.00253)	9 (2)	0.259*	0.822	0.615
TEN	24	0.000	0.000	(±0.000)	0.00000	(±0.00000)	1	29	5.349	0.743	(±0.040)	0.03475	(±0.00176)	10 (1)	−0.210*	0.744	0.897
KNY	23	2.354	0.668	(±0.057)	0.00359	(±0.00101)	4	19	8.145	0.828	(±0.044)	0.03608	(±0.00144)	12 (4)	−0.018	0.828	0.842
PE	20	1.158	0.195	(±0.115)	0.00532	(±0.00304)	3	12	14.83	0.953	(±0.029)	0.03889	(±0.00212)	17 (3)	0.040	0.953	0.917
NC	23	3.323	0.692	(±0.085)	0.00374	(±0.00094)	6 (6)	18	8.927	0.789	(±0.065)	0.02859	(±0.00429)	13 (8)	0.586*	0.792	0.333
SC	25	2.976	0.710	(±0.060)	0.00491	(±0.00046)	5 (2)	18	7.277	0.807	(±0.050)	0.02797	(±0.00251)	11 (1)	0.022	0.807	0.790
CAL	11	3.000	0.800	(±0.075)	0.01684	(±0.00270)	4 (1)	11	5.000	0.818	(±0.049)	0.04023	(±0.00248)	6	−0.236	0.818	1.000
BRA	19	1.818	0.503	(±0.113)	0.01100	(±0.00294)	3	17	6.882	0.775	(±0.052)	0.03290	(±0.00199)	10 (2)	−0.301*	0.775	1.000
AM	24	0.458	0.083	(±0.005)	0.00294	(±0.00264)	2	22	3.140	0.596	(±0.058)	0.01101	(±0.00118)	5	0.242	0.596	0.455
WAK	25	1.690	0.527	(±0.084)	0.00212	(±0.00035)	3	24	7.863	0.806	(±0.043)	0.03334	(±0.00222)	14 (3)	−0.035	0.806	0.833
OKI	24	1.717	0.424	(±0.112)	0.00162	(±0.00042)	3	16	4.972	0.766	(±0.044)	0.03892	(±0.00176)	7	−0.233	0.766	0.938
MIS	25	1.361	0.347	(±0.108)	0.01043	(±0.00309)	3	22	6.178	0.780	(±0.044)	0.03019	(±0.00208)	10 (1)	−0.230*	0.780	0.955
SKS	24	2.437	0.663	(±0.065)	0.00175	(±0.00033)	4 (1)	24	4.536	0.714	(±0.044)	0.03725	(±0.00128)	8 (1)	−0.414*	0.714	1.000
HK	24	2.891	0.692	(±0.065)	0.00269	(±0.00061)	5 (1)	13	9.614	0.834	(±0.044)	0.02363	(±0.00199)	12 (5)	−0.177	0.855	1.000
MED	40	3.000	0.314	(±0.091)	0.00226	(±0.00073)	4 (1)	39	5.377	0.554	(±0.058)	0.01833	(±0.00176)	7 (1)	0.494*	0.554	0.282
ATL	128	9.419	0.759	(±0.034)	0.01373	(±0.00098)	17 (12)	124	17.60	0.852	(±0.015)	0.03269	(±0.00089)	34 (20)	0.155*	0.858	0.726
PAC	157	4.544	0.768	(±0.011)	0.01380	(±0.00076)	8 (3)	132	13.55	0.803	(±0.016)	0.03200	(±0.00078)	27 (10)	−0.067*	0.809	0.864
IND	43	3.930	0.717	(±0.038)	0.01566	(±0.00085)	5	31	21.00	0.883	(±0.027)	0.03683	(±0.00103)	22 (8)	0.013	0.883	0.871
**Total**	368	8.124	0.810	(±0.010)	0.01348	(±0.00057)	22	315	16.32	0.820	(±0.012)	0.03214	(±0.00059)	61	0.098*	**0.824**	**0.743**

Number of individuals analyzed per population (*N*). Allelic richnesstandarized across populations (r), Gene (*Hd*) and nucleotidic (*π*) diversity, and their corresponding standard deviations in brackets. Number of alleles per population (*Nh*), with private alleles shown in brackets. Inbreeding coefficient (*F_is_*) for *ANT*. *Asterisks* represent significant coefficients at *P*<0.05. *H_exp_* represents the expected heterozygosity and *H_obs_* represents the observed heterozygosity.

**Figure 2:**
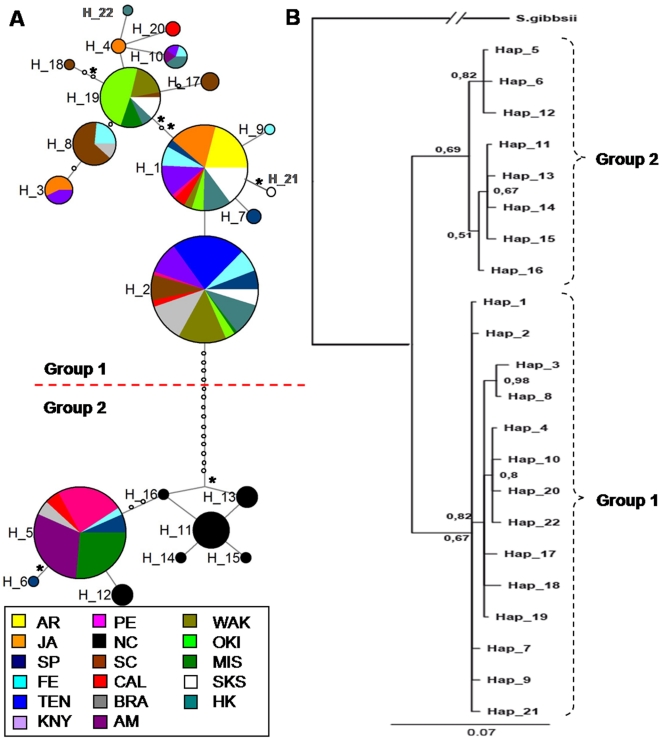
Network and phylogeny for *COI*. a) Median-joining haplotype network for *Styela plicata* using *COI* results. Area of *circles* is proportional to the number of individuals found for each haplotype. Partitions inside the *circles* represent the proportion of each population within each haplotype. Small circles represent missing haplotypes. Lines between circles represent one mutational step and non-synonymous substitutions are indicated with an asterisk; b) Phylogeny of partial *COI* gene sequences using Bayesian inference. The congeneric species *Styela gibbsii* was used as an outgroup. Posterior probabilities are indicated when >0.5.

Jost's adjusted estimator (D_est_) was used to assess the allelic differentiation between populations for each marker, showing high values of differentiation (mean D_est_ = 0.660). The *COI* data revealed high differentiation between many population-pairs, as 88 comparisons out of 136 resulted in significant differences after correction for multiple comparisons (Table 3). For instance, the North Carolina population had no alleles in common with any other population (Fig. 2), and many other populations (e.g. Port Elizabeth, Manly, Misaki, Okinawajima) also differed considerably in their allele composition. No particular pattern was found for the only population collected from natural substratum (Sakushima Island, SKS), which was significantly different from half of the remaining populations.

**Table 3:** Jost's D_est_ population differentiation statistic between populations of *Styela plicata* for the *COI* (upper diagonal) and *ANT* (lower diagonal) markers.

AR	JA	SP	FE	TEN	KNY	PE	NC	SC	CAL	BRA	AM	WAK	OKI	MIS	SKS	HK
	0.067	**0.753**	**0.483**	**0.948**	0.366	**0.938**	**1**	**0.973**	0.521	**0.951**	**1**	**0.844**	**0.832**	**0.997**	0.162	**0.442**
0		**0.76**	**0.452**	**1**	0.299	**0.944**	**1**	**1**	0.481	**1**	**1**	**0.888**	**0.841**	**1**	0.132	**0.439**
0.036	0.082		0.114	0.381	0.219	**0.504**	**1**	**0.52**	0.086	0.129	**0.575**	0.269	**0.841**	**0.502**	0.458	0.177
0.032	0.129	0.015		**0.45**	0.05	**0.767**	**1**	0.241	0.184	0.246	**0.835**	0.311	**0.804**	**0.793**	0.185	0.032
**0.49**	**0.49**	0.346	**0.486**		0.351	**0.942**	**1**	**0.506**	**0.702**	0.091	**1**	0.135	**0.842**	**0.952**	**0.666**	0.303
0.281	0.289	0.116	0.258	0.058		**0.923**	**1**	**0.557**	0.325	0.29	**0.997**	0.238	**0.774**	**0.965**	0.101	−0.03
**0.522**	**0.567**	0.342	0.391	0.318	0.138		**1**	**0.969**	0.312	**0.656**	0.003	**0.941**	**0.981**	0.015	**0.93**	**0.925**
**0.978**	**0.99**	**0.992**	**0.945**	**0.897**	**0.832**	**0.715**		**1**	**1**	**1**	**1**	**1**	**1**	**1**	**1**	**1**
0.098	0.157	0	0.042	0.213	0.086	0.231	**0.978**		**0.771**	0.333	**1**	0.409	**0.771**	**0.946**	**0.732**	**0.493**
0.35	0.358	0.176	0.314	0	0	0.134	**0.923**	0.07		0.44	0.386	**0.6**	**0.857**	0.316	0.338	0.338
0.269	0.267	0.125	0.273	0.018	0	0.274	**0.97**	0.074	0		**0.716**	0.105	**0.842**	**0.662**	**0.639**	0.229
0.134	0.113	0.099	0.189	**0.461**	0.319	**0.509**	**1**	0.09	0.297	0.284		**1**	**1**	0.027	**1**	**0.994**
**0.538**	**0.543**	0.353	**0.51**	0	0.06	0.3	**0.966**	0.212	0	0.02	**0.482**		0.432	**0.876**	0.425	0.128
0.261	0.273	0.161	0.274	0.084	0.015	0.117	**0.875**	0.106	0	0.025	0.321	0.142		**0.798**	**0.503**	**0.637**
**0.479**	**0.499**	0.315	**0.457**	0	0.066	0.281	**0.95**	0.157	0	0.025	**0.427**	0	0.107		**0.925**	**0.935**
0.22	0.21	0.128	0.259	0.051	0.001	0.273	**0.937**	0.082	0	0	0.248	0.093	0	0.071		0.101
**0.525**	**0.636**	**0.585**	0.388	**0.826**	**0.754**	**0.789**	**0.94**	**0.604**	**0.759**	**0.758**	**0.718**	**0.822**	**0.774**	**0.777**	**0.761**	

Values in bold represent significant comparisons after FDR correction (see text).

The results of the hierarchical AMOVA showed higher within population variability (58.41%) than the one between populations (41.59%, *P*<0.001, Table 4). AMOVA analyses performed by grouping populations according to their oceanic basin revealed that most of the genetic diversity was due to variability within populations (56.97%, *P*<0.001), and among populations within basins (34.36%, *P*<0.001). However, no significant differences in genetic structure were detected between basins (8.67%, *P* = 0.055 for *COI*; Table 4). Accordingly, the Mantel test showed no correlation between genetic differentiation and geographical distance between populations (*r* = 0.00009, *P* = 0.434).

**Table 4:** Analysis of the molecular variance (AMOVA) for the *COI* and *ANT* genetic markers.

Source of variation	*df*	Sum of squares	Variance components	Variation (%)	*P* value	Fixation indices
a) *COI*						
AMOVA without groups						
Among populations without groups	16	63.536	0.17255 Va	41.59*	0.000	F_ST_: 0.41589
Within populations	351	85.064	0.24235 Vb	58.41		
Total	367	148.601	0.4149			
AMOVA between basins						
Among groups	3	19.279	0.03690 Va	8.67	0.055	F_CT_ : 0.08673
Among populations within groups	13	44.257	0.14618 Vb	34.36*	0.000	F_SC_ : 0.37624
Within populations	351	85.064	0.24235 Vc	56.97*	0.000	F_ST_ : 0.43034
Total	367	148.601	0.42543			
b) *ANT*						
AMOVA without groups						
Among populations without groups	16	28.988	0.03892 Va	9.40*	0.000	F_ST_: 0.09397
Within populations	613	230.022	0.37524 Vb	90.6		
Total	629	259.01	0.41416			
AMOVA between basins						
Among groups	3	7.806	0.00670 Va	1.61	0.127	F_CT_ : 0.01610
Among populations within groups	13	21.182	0.03412 Vb	8.20*	0.000	F_SC_ : 0.08336
Within populations	613	230.022	0.37524 Vc	90.19*	0.000	F_ST_ : 0.09812
Total	629	259.01	0.41606			

Analyses are presented for the total of populations without grouping, and pooling populations from the same oceanic basin together (Mediterranean, Atlantic, Pacific and Indian). Va, Vb and Vc are the associated covariance components. F_SC_, F_ST_ and F_CT_ are the *F-*statistics.

Overall, neutrality tests were not significant (Table 5), and hence did not support any lack of equilibrium due to selection or population size changes at any level (either partitioned by populations or oceanic basins). The only exceptions encountered were for the Australian population of Manly (AM), with significantly negative Tajima's *D* values, and for Sakushima and the Group 1 of haplotypes (see below), with a significant raggedness index (Table 5).

**Table 5:** Demographic parameters of *S. plicata* populations for each genetic marker (*COI* and *ANT*), calculated for each population and samples grouped by basin and by group (1 and 2 for *COI*, and A and B for *ANT*).

	*COI*				*ANT*			
	*D*	*Fs*	*R_2_*	*r*	*D*	*Fs*	*R_2_*	*r*
AR	−1.16439	−0.879	0.218	0.650	1.29064	2.347	0.169	0.243
JA	0.74648	3.941	0.173	0.462	0.25898	2.715	0.126	0.345
SP	2.15635	6.162	0.229	0.103	0.59380	0.232	0.143	0.077
FE	−0.83585	3.033	0.104	0.112	1.04251	−0.535	0.170	0.032
TEN	0.00000	0.000	0.000	0.000	2.32335	5.011	0.187	0.190
KNY	−0.27356	2.391	0.123	0.149	2.15146	1.718	0.196	0.068
PE	−1.29958	5.371	0.090	0.658	1.83362	−4.076*	0.197	0.021
NC	−0.14467	0.419	0.124	0.127	−0.15150	−0.920	0.112	0.044
SC	0.52180	2.497	0.153	0.348	0.63874	−0.198	0.141	0.140
CAL	1.81929	6.420	0.239	0.155	2.46514	5.670	0.229	0.119
BRA	0.55113	9.699	0.164	0.483	0.94915	0.814	0.152	0.101
AM	−2.53406**	5.308	0.200	0.854	0.83652	2.602	0.149	0.366
WAK	1.64264	2.196	0.220	0.384	2.48268	0.904	0.201	0.066
OKI	0.64968	1.430	0.169	0.360	3.02590	6.494	0.235	0.215
MIS	0.82576	10.821	0.163	0.578	1.06354	1.146	0.152	0.150
SKS	0.05885	0.400	0.136	0.043*	3.17433	7.094	0.226	0.244
HK	0.13328	0.478	0.137	0.069	0.50405	−0.338	0.141	0.046
MED	−0.71549	1.657	0.087	0.482	1.01299	2.380	0.139	0.286
ATL	1.10126	3.816	0.125	0.109	1.02151	−7.404*	0.114	0.046
PAC	2.66373	15.635	0.172	0.103	1.72095	−2.885	0.136	0.081
IND	2.31343	−0.246	0.108	0.033	2.44640	−1.956	0.190	0.029
Group 1(A)	−0.84647	−2.032	0.054	0.024*	−0.04229	−11.460**	0.083	0.066
Group 2(B)	−0.53974	−0.488	0.075	0.360	−0.29695	−6.598	0.067	0.140

*Asterisks* represent significant results:

*P<0.05;

**P<0.002.

Tajima's *D*, Fu's *F*s statistic, Ramos-Onsins & Rozas's statistic (*R_2_*), and the raggedness index (*r*).

The network obtained for the *COI* gene (Fig. 2a) revealed two divergent lineages (hereafter called Group 1 and Group 2) separated by 15 mutational steps and without any intermediate haplotype in between. McDonald-Kreitman (MK) test of neutrality showed that there were no differences between proportions of silent and replacement sites within and between these two groups (*P* = 0.64). Sequences from both Group 1 and 2 are found in all basins and coexist in most populations; except for the absence of Group 2 in the Mediterranean. Judging by their high frequency, wide geographical distribution, and central position in the network, H_2 may be the ancestral haplotype of Group I. No clear result was obtained for group 2, as the most abundant haplotype (H_5) occupied a distal position within the group. (Fig. 2a). The BI tree reconstructed with *COI* haplotypes showed two moderately supported clades exhibiting 3.27% sequence divergence among them (Fig. 2b). These two clades matched exactly with Group 1 and 2 described for the *COI* network (Fig. 2a). Haplotype H_2 (inferred as ancestral) held a basal position within Group 1, while no evidence for a basal haplotype or group of haplotypes was found for Group 2.

### Nuclear gene

For the *ANT* gene, we obtained 315 sequences of 220 bp. The *ANT* fragment targeted here includes an intron in many metazoans [63]. However, in our case, all sequences could be translated to amino acids and final sequence length was in accordance with what has been found for species without an intron in this position [63]. Our resulting dataset contained 80 homozygotes, which allowed a reliable reconstruction of the gametic phase of the heterozygotes (>95% confidence). No evidence was detected for recombination within our sequences. In total we obtained 61 alleles (Tables S2 and S3), 34 in the Atlantic (20 of which were exclusive to this basin) and 27 in the Pacific (Table 2). A deletion of 22 amino acids was found in 5 alleles (Table S2). Once more, the Mediterranean showed the lowest number of alleles (7, of which only one was private). Mean and total haplotype diversity (*Hd*) were 0.761 (±0.011 SD) and 0.820 (±0.012 SD), respectively. Mean nucleotide diversity was 0.0295 (±0.008 SD), while total nucleotide diversity (*π*) was 0.0321 (±0.0006 SD). Gene and nucleotide diversity did not differ between basins, except for the Mediterranean (Table 2). The South African populations of Knysna (KNY) and Port Elizabeth (PE) showed the highest values for genetic diversity, followed by most Pacific populations and some Atlantic ones (Table 2). Port Elizabeth (PE) was also the population showing the highest allelic richness (14.830) followed by Hong Kong (HK, 9.614), North Carolina (NC, 8.927) and Knysna (KNY, 8.145). As found for the mitochondrial gene, the lowest value of allelic richness corresponded to Manly (AM, 3.140). Low values were also retrieved for the Mediterranean populations of Javea (JA, 3.307) and Arenys de Mar (AR, 3.733). Comparisons between basins indicated that the Indian Ocean had the highest allelic richness, while the Mediterranean had the lowest (Table 2). Eight populations had less heterozygotes than expected, five of which (Arenys de Mar, Javea, San Fernando, Ferrol and North Carolina) deviated significantly from Hardy-Weinberg equilibrium (significant F_is_ values). Interestingly, 9 populations had an excess of heterozygotes (and negative F_is_), and in 4 of them (Tenerife, Brasil, Misaki, Sakushima) these inbreeding coefficients were significant. Per basins, there was a heterozygote deficit in all populations except for the Pacific, and this deficit was most marked for the Mediterranean group of populations (0.282 *H_obs_ vs.* 0.554 *H_exp_*).

Jost's adjusted estimator showed lower values of differentiation for the nuclear intron *ANT* (mean D_est_ = 0.324) than for the mitochondrial *COI*. D_est_ values obtained for the *ANT* gene revealed fewer significant differences in pair-wise comparisons (45 out of 136). As before, the North Carolina population was significantly different from all the others (Table 3). Interestingly, the Sakushima population (on natural substratum) only differed from the North Carolina and Hong Kong populations.

The hierarchical AMOVA analyses showed that most of the observed variability was found within populations (90.6%), and only a small but significant 9.4% (p<0.001) of variability was found among these populations (Table 4). When grouping populations according to their oceanic basins, AMOVA analyses' results were similar to those found for the mitochondrial marker. Most of the genetic diversity was due to variability within populations (90.19%, *P*<0.001), and among populations within basins (8.20%, *P*<0.001). No significant differences in genetic structure were detected between basins (1.61%, *P* = 0.127 ; Table 4). As found for *COI*, the Mantel test showed no correlation between genetic differentiation and geographical distance between populations (*r* = 0.000001, *P* = 0.243). Regarding the neutrality test, the same trend of *COI* was observed for *ANT*, with most tests being non-significant. However, Fu's *F_s_* were significant for the Atlantic Ocean and the Port Elizabeth population (Table 5).

**Figure 3:**
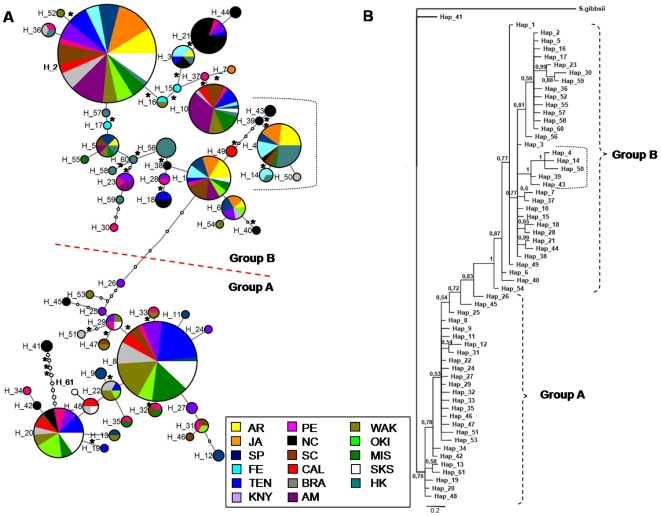
Network and phylogeny for *ANT*. a) Median-joining allele network for *Styela plicata* using *ANT* results. Area of *circles* is proportional to the number of individuals found for each allele. Partitions inside the *circles* represent the proportion of each population within each allele. Small circles represent missing alleles. Lines between circles represent one mutational step and non-synonymous substitutions are indicated with an asterisk; b) Phylogeny of partial *ANT* gene sequences using Bayesian inference. The congeneric species *Styela gibbsii* was used as an outgroup. Posterior probabilities are indicated when >0.5. The dot line mark the clade corresponding to sequences with a 22 amino acid deletion.

Network analyses showed a considerable amount of loops that were unambiguously resolved following coalescent rules (Fig. 3a). None of these loops affected the main structures shown in the network. However, the relationship among alleles should be considered with caution and no clear ancestral allele could be reliably designated. Although less divergent than with the *COI* data, the *ANT* network also showed a distinction in two groups of sequences separated by 4 mutational steps (Fig. 3a). None of these four mutations corresponded to non-synonymous changes. Finally, the 22 amino acids deletion found in 5 alleles (H_4, H_14, H_39, H_43, H_50) was also retrieved (represented by a dot line in Fig. 3a). McDonald-Kreitman neutrality tests could not be performed between these groups, as there was no fixed difference between them. BI analysis showed that one of the groups (hereafter called Group A) occupied a basal position within the resulting tree, while a second group (Group B) formed a monophyletic, derived clade supported by a posterior probability of 1 (Fig. 3b). Within group B, the five alleles with a 22 amino acid deletion also formed a monophyletic clade (posterior probability = 1; Fig. 3b). When the sequence fragment corresponding to the deletion was removed from the analyses, these 5 alleles still grouped together, indicating that their phylogenetic relationship was independent from the indel presence. The alleles containing the deletion were found in all studied basins, not showing any apparent geographic pattern (Table S2, Figure 3a).

The private allele H_41 from North Carolina appeared genetically distinct from all the others in both the network and the BI analyses (Fig. 3a). This sample was re-extracted and sequenced *de novo*, but the same resulting sequence was obtained. The Mediterranean populations only presented alleles from Group A of *ANT*, while the remaining populations presented alleles from both groups (especially, those populations from the Pacific Ocean). This pattern explains the lower genetic diversity found in the Mediterranean basin compared with that of the other oceans. Group B seems to be a highly successful derived clade that has spread in most populations. Interestingly, in all localities in which there was an excess of heterozygotes (negative F*_is_*), there was also a higher than expected proportion of individuals having one allele of each group (A or B; 0.75 observed *vs.* 0.49 expected frequency). This is especially noteworthy in the Pacific populations, where we found twice the number of “mixed” genotypes than expected. The only exception was for North Carolina, which had a significant deficit of heterozygotes and less than expected genotypes with an allele from each group.

**Figure 4:**
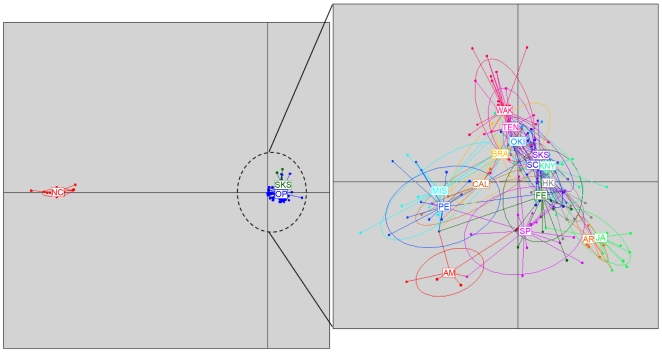
Discriminant analysis of principal components (DAPC). Left: plot of the first two principal components obtained in the DAPC analysis considering three groups: the North Carolina population (NC), the Sakushima Island population (SKS) and other populations (OP). Right: plot of the DAPC results analyzing all populations as individual groups, except North Carolina, which was not analyzed (see text). Population codes as in Table 1. Labels are placed at the centre of dispersion for each group, further delineated by inertia ellipses. Dots represent individuals.

Finally, DAPC analyses were performed combining results obtained for *COI* and *ANT*. In order to avoid cluttering of populations, a first DAPC was performed with 3 groups: the North Carolina population (significantly different from the rest in previous analyses), the Sakushima population (the only natural substratum population) and the remaining populations. The PCA components retained explained 98.6% of the total variance observed. The scatterplot of the first two components of the DA (Fig. 4) showed that the first axis separates North Carolina from the rest, which form a tight cluster, while the second axis slightly sets apart the Sakushima population, although with a clear overlap of the inertia ellipses. We then repeated the analysis removing the North Carolina population and considering all populations as separate groups. 99.2% of the total variance was explained by the retained components of the PCA. The populations appeared mixed in the space of the first two axes of the discriminant analysis (Fig. 4), although the first axis separated slightly Misaki, Port Elizabeth and Manly on one extreme, and the two Mediterranean populations at the other end. The rest of the populations clustered tightly together, with the natural substratum population (Sakushima) appearing in a central position.

## Discussion

Several remarkable features emerged from the recovered distribution of the genetic variability. First, there is a divergence in lineages for both markers, each featuring two groups of sequences. Second, the genetic pool is well mixed at the basin level, with little or no phylogeographic signal remaining. Third, many population pairs are genetically different, regardless of the geographic distance among them. Finally, there seems to be an effect of selection on the genetic makeup of this species, as illustrated by the highly divergent population of North Carolina and the intra-individual distribution of both groups of *ANT* sequences.

The most parsimonious explanation for the presence of two groups of sequences for *COI* (group 1 and 2) and *ANT* (group A and B) is that they have arisen concomitantly in a past fragmentation event within the native area of the species. We cannot, however, exclude an independent origin of these genetic splits. At present, the distribution of the groups obtained with the two markers is totally unrelated. Sequences of the Group A for *ANT* were found in ascidians having mitochondrial sequences of both lineages (Groups 1 and 2), and in direct proportion to their relative abundances. The same trend was observed for individuals having sequences of Group B for *ANT* (Table S3). If the differentiation of *ANT* and *COI* in different lineages occurred simultaneously in allopatric regions, the link between these markers was lost long ago. Mitochondrial genes are inherited maternally, while nuclear genes can be shuffled repeatedly through sexual reproduction. Thus, the lack of congruence found in the distribution of both markers could be due to frequent contact between individuals from different lineages coupled with genetic drift. A greater sensitivity of mitochondrial genes to genetic drift has been previously reported [88], and may explain the differences observed between mitochondrial and nuclear markers [e.g. 88]–[90]. In addition, no geographic pattern was observed in the distributions of the lineages observed for both markers. Even in the putative native area of *S. plicata* (NW Pacific), we found sequences of the two groups of *COI* and *ANT* in the same populations and, for *ANT*, even in the same individual.

Barros et al. [54] found nine *COI* haplotypes for *Styela plicata*, 8 belonging to our Group 1 and one to our Group 2. Based on this divergent haplotype, these authors suggested that there could be a cryptic species within what is known as *Styela plicata*. Our results did not lend support to this hypothesis, as the nuclear marker showed a distribution unrelated to these two groups of mitochondrial sequences. Furthermore, when comparing our mitochondrial sequences with other species of the genus, the resulting genetic divergence was much higher than that found between our two *COI* groups (3.27% between our groups, 21.12% between *S. plicata* and *S. gibbsii*; 22.7% with *S. clava*, and 20% with *S. montereyensis*). The divergent sequences of *S. plicata* reported from Australia (Lake Conjola) by Pérez-Portela et al. [107] (GenBank accession numbers FJ528633-34 for *COI* and FH897323 for 18S rRNA) were likely the result of sample mislabelling (Pérez-Portela, *pers. comm*.). We sequenced 4 further specimens from the same locality and verified that they all had typical *S. plicata COI* sequences (i.e., Haplotype 5).

Although the native range of *Styela plicata* is not known with certainty, the prevailing hypothesis is that it comes from the NW Pacific area [36], [54]. *S. plicata* would have then dispersed to other tropical and warm-water regions by ship fouling, likely since the early transoceanic navigation times [36]. Our results indicated that at present the genetic pool of *S. plicata* is well mixed among basins, with most genetic variability found within populations. Moreover, high genetic variability and the putatively most ancient alleles have not only been found in the NW Pacific populations (e.g. Sakushima, Hong Kong) but also in other oceanic basins (e.g. North East Pacific, Atlantic and Indian Ocean; see also David et al. [91]). Thus, we could not find any clear genetic signal in favour (or against) the hypothesis on the NW Pacific origin of this species. The only potential trend observed in our data was for the Mediterranean basin. The Mediterranean populations presented the lowest values for all diversity indexes, and only displayed group 1 for *COI* and group B for *ANT*. However, these findings should be interpreted with caution, as only two Mediterranean localities were included in this study. Lack of resolution for assessing native areas was also found in studies with other ascidian species that are believed to be ancient colonizers (e.g. *Ciona intestinalis* [38]). On the other hand, species that have spread more recently still have a genetic signature of their introduction history (e.g. *Botryllus schlossei* [41], [42], *Microcosmus squamiger* [92], *Styela clava* [45]).

Long-distance dispersal of introduced marine species across oceans probably occurs via major shipping routes while further spread at a local scale may take place through local traffic and recreational boating [13], [34], [42], [91], [93]. Our results indicate that many populations of *S. plicata* are well differentiated from others in terms of allele frequencies. This observation is in agreement with results obtained for other ascidians inhabiting harbours and marinas [37], [41], [44], [ but see 38 for an exception]. As expected when anthropogenic transport is the vector of dispersal, genetic differentiation among *S. plicata* populations was unrelated to geographic distance. Some distant populations (e.g. Hong Kong and Ferrol) were genetically similar, while closer populations such as Knysna and Port Elizabeth (South Africa) were significantly divergent. The stochasticity of main transport events through international ship traffic could determine the observed patterns among basins. However, our sampling design was inappropriate to assess the degree of connectivity among closely located populations (i.e. post-border dispersion, [34]). Thus, it still remains necessary to evaluate the role of small-scale processes in colonization dynamics, and to assess the importance of recreational boating in spreading introduced species.

Low genetic diversity caused by a founder effect or a bottleneck is not always the benchmark for introductory events [28], [94], [95]. In fact, recurrent introductions typically lead to highly diverse populations, especially if they receive migrants from native populations that are genetically structured [26], [30], [44], [96], [97]. Here, we found that genetic diversity indexes varied according to the studied population, with overall values ranging from moderate to high for both markers. Some exceptions were these populations where only one or two mitochondrial haplotypes were present (i.e. Arenys de Mar, Tenerife, Manly).

Besides recurrent introductions through ship transport, population differentiation could also be due to selection. Here, we found uneven abundances for each major group obtained for *COI* (Group 1 and 2) and *ANT* (Group A and B). For *COI*, haplotypes from Group 1 were considerably more frequent and diverse than haplotypes from Group 2. It is possible that these groups stand for differential adaptive capabilities of the individuals to stressful environments. This adaptive capability does not need to be directly linked to our studied gene (non-significant McDonald-Kreitman test), but to other mitochondrial genes. Differential adaptation to environmental factors (e.g., temperature, salinity) of mitochondrial sequences within one species is not a rare phenomenon, and has been described in many species [98]–[104].

For the *ANT* gene, selection may be favouring heterozygotes that have an allele of each group (A and B). In fact, the excess of heterozygotes found in most populations is due to the number of individuals with an allele each of A and B. Accordingly, the number of individuals with both alleles from the same group (A or B) was lower than expected. Homozygotes for the basal Group A occurred ca. 5 times less than expected based on allele frequencies. Thus, it is possible that populations that originally had only one group of *ANT* sequences were seeded with arriving individuals featuring the other group. The mingling of both groups may have favoured the heterozygotes with an allele from each group, and if this combination had an adaptive value, enhanced the fitness of those individuals. As for the *COI* lineages, this new adaptive capability to the environment is not necessarily linked to the *ANT* gene itself. Admixture between lineages can foster the emergence of novel genetic combinations with different physiological attributes and invasive characteristics [30]. In contrast to our results, solitary ascidians inhabiting artificial structures usually have a general deficit of heterozygotes [38], [44], [105].

Early invasions should not be considered “naturalized,” rather, their impacts, potential for further spread, and degree of integration in local processes and interactions should be assessed. A throughout knowledge of introduced species is required to understand and interpret the present-day structure, function, and conservation of marine communities [7], [35], [36]. Our genetic study of an ancient wanderer has uncovered signatures of deep divergences and recent mixing, with a phylogeographic signal mostly blurred. Current evolutionary processes may include adaptive changes and low and stochastic connectivity among established populations. More studies on *S. plicata*'s biological cycle, interactions with other marine species, and local-scale genetic structure are necessary to understand the biology, ecology and post-border dispersal of this species and prevent ecosystem alterations.

## Supporting Information

Table S1 Haplotype frequencies observed for the *COI* gene. Numbers in bold are private haplotypes.
(DOC)

Table S2 Allele frequencies observed for the *ANT* gene. Sequences with a 22 amino acid deletion are indicated with an asterisk.
(DOC)

Table S3 *ANT* allelic phase and *COI* haplotypes for each individual analyzed.
(DOC)
