# Supplementary material for: The Whereabouts of an Ancient Wanderer: Global Phylogeography of the Solitary Ascidian Styela plicata
Source: PLoS One. 2011 Sep 23;6(9):e25495. doi: 10.1371/journal.pone.0025495 (PMC3179514; doi:10.1371/journal.pone.0025495)
Supplement: Table S1 — Haplotype frequencies observed for the COI gene. Numbers in bold are private haplotypes. (DOC) [file pone.0025495.s001.doc]

|  | AR | JA | SP | FE | TEN | KNY | PE | NC | SC | CAL | BRA | AM | WAK | OKI | MIS | SKS | HK |
| --- | --- | --- | --- | --- | --- | --- | --- | --- | --- | --- | --- | --- | --- | --- | --- | --- | --- |
| H_1 | 0.95 | 0.7 | 0.125 | 0.2857 | 0 | 0.391 | 0.05 | 0 | 0 | 0.273 | 0 | 0 | 0.08 | 0.125 | 0 | 0.5 | 0.333 |
| H_2 | 0.05 | 0 | 0.375 | 0.3333 | 1 | 0.435 | 0.05 | 0 | 0.32 | 0.182 | 0.684 | 0 | 0.64 | 0.125 | 0.04 | 0.292 | 0.458 |
| H_3 | 0 | 0.2 | 0 | 0 | 0 | 0.13 | 0 | 0 | 0 | 0 | 0 | 0 | 0 | 0 | 0 | 0 | 0 |
| H_4 | 0 | **0.1** | 0 | 0 | 0 | 0 | 0 | 0 | 0 | 0 | 0 | 0 | 0 | 0 | 0 | 0 | 0 |
| H_5 | 0 | 0 | 0.313 | 0.095 | 0 | 0 | 0.9 | 0 | 0 | 0.364 | 0.211 | 0.958 | 0 | 0 | 0.8 | 0 | 0 |
| H_6 | 0 | 0 | **0.063** | 0 | 0 | 0 | 0 | 0 | 0 | 0 | 0 | 0 | 0 | 0 | 0 | 0 | 0 |
| H_7 | 0 | 0 | **0.125** | 0 | 0 | 0 | 0 | 0 | 0 | 0 | 0 | 0 | 0 | 0 | 0 | 0 | 0 |
| H_8 | 0 | 0 | 0 | 0.19 | 0 | 0 | 0 | 0 | 0.44 | 0 | 0.105 | 0 | 0 | 0 | 0 | 0 | 0 |
| H_9 | 0 | 0 | 0 | **0.048** | 0 | 0 | 0 | 0 | 0 | 0 | 0 | 0 | 0 | 0 | 0 | 0 | 0 |
| H_10 | 0 | 0 | 0 | 0.048 | 0 | 0.043 | 0 | 0 | 0 | 0 | 0 | 0.042 | 0 | 0 | 0 | 0 | 0.083 |
| H_11 | 0 | 0 | 0 | 0 | 0 | 0 | 0 | **0.522** | 0 | 0 | 0 | 0 | 0 | 0 | 0 | 0 | 0 |
| H_12 | 0 | 0 | 0 | 0 | 0 | 0 | 0 | **0.174** | 0 | 0 | 0 | 0 | 0 | 0 | 0 | 0 | 0 |
| H_13 | 0 | 0 | 0 | 0 | 0 | 0 | 0 | **0.174** | 0 | 0 | 0 | 0 | 0 | 0 | 0 | 0 | 0 |
| H_14 | 0 | 0 | 0 | 0 | 0 | 0 | 0 | **0.043** | 0 | 0 | 0 | 0 | 0 | 0 | 0 | 0 | 0 |
| H_15 | 0 | 0 | 0 | 0 | 0 | 0 | 0 | **0.043** | 0 | 0 | 0 | 0 | 0 | 0 | 0 | 0 | 0 |
| H_16 | 0 | 0 | 0 | 0 | 0 | 0 | 0 | **0.043** | 0 | 0 | 0 | 0 | 0 | 0 | 0 | 0 | 0 |
| H_17 | 0 | 0 | 0 | 0 | 0 | 0 | 0 | 0 | **0.12** | 0 | 0 | 0 | 0 | 0 | 0 | 0 | 0 |
| H_18 | 0 | 0 | 0 | 0 | 0 | 0 | 0 | 0 | **0.04** | 0 | 0 | 0 | 0 | 0 | 0 | 0 | 0 |
| H_19 | 0 | 0 | 0 | 0 | 0 | 0 | 0 | 0 | 0.08 | 0 | 0 | 0 | 0.28 | 0.75 | 0.16 | 0.167 | 0.083 |
| H_20 | 0 | 0 | 0 | 0 | 0 | 0 | 0 | 0 | 0 | **0.182** | 0 | 0 | 0 | 0 | 0 | 0 | 0 |
| H_21 | 0 | 0 | 0 | 0 | 0 | 0 | 0 | 0 | 0 | 0 | 0 | 0 | 0 | 0 | 0 | **0.042** | 0 |
| H_22 | 0 | 0 | 0 | 0 | 0 | 0 | 0 | 0 | 0 | 0 | 0 | 0 | 0 | 0 | 0 | 0 | **0.042** |
