# Supplementary material for: The Whereabouts of an Ancient Wanderer: Global Phylogeography of the Solitary Ascidian Styela plicata
Source: PLoS One. 2011 Sep 23;6(9):e25495. doi: 10.1371/journal.pone.0025495 (PMC3179514; doi:10.1371/journal.pone.0025495)
Supplement: Table S2 — Allele frequencies observed for the ANT gene. Sequences with a 22 amino acid deletion are indicated with an asterisk. (DOC) [file pone.0025495.s002.doc]

|  | AR | JA | SP | FE | TEN | KNY | PE | NC | SC | CAL | BRA | AM | WAK | OKI | MIS | SKS | HK |
| --- | --- | --- | --- | --- | --- | --- | --- | --- | --- | --- | --- | --- | --- | --- | --- | --- | --- |
| H_1 | 0.158 | 0.1 | 0.088 | 0.115 | 0.017 | 0 | 0.042 | 0 | 0.132 | 0 | 0 | 0.068 | 0 | 0.063 | 0.045 | 0.02 | 0 |
| H_2 | 0.579 | 0.7 | 0.441 | 0.385 | 0.276 | 0.342 | 0.167 | 0 | 0.395 | 0.318 | 0.382 | 0.568 | 0.229 | 0.375 | 0.25 | 0.449 | 0.115 |
| H_3 | 0.026 | 0 | 0 | 0.115 | 0.017 | 0 | 0 | 0.056 | 0 | 0 | 0 | 0 | 0 | 0 | 0 | 0 | 0.038 |
| H_4* | 0.184 | 0.125 | 0.059 | 0.154 | 0 | 0 | 0 | 0.028 | 0.053 | 0 | 0 | 0 | 0 | 0 | 0.023 | 0 | 0.308 |
| H_5 | 0.026 | 0 | 0.059 | 0 | 0 | 0 | 0 | 0 | 0.026 | 0 | 0 | 0.023 | 0.021 | 0 | 0.023 | 0 | 0.038 |
| H_6 | 0.026 | 0.05 | 0.059 | 0 | 0 | 0.053 | 0 | 0 | 0 | 0 | 0.029 | 0 | 0 | 0.031 | 0 | 0 | 0 |
| H_7 | 0 | **0.025** | 0 | 0 | 0 | 0 | 0 | 0 | 0 | 0 | 0 | 0 | 0 | 0 | 0 | 0 | 0 |
| H_8 | 0 | 0 | 0.059 | 0 | 0.414 | 0.237 | 0.083 | 0 | 0.132 | 0.273 | 0.294 | 0 | 0.375 | 0.219 | 0.386 | 0.286 | 0 |
| H_9 | 0 | 0 | **0.059** | 0 | 0 | 0 | 0 | 0 | 0 | 0 | 0 | 0 | 0 | 0 | 0 | 0 | 0 |
| H_10 | 0 | 0 | 0.059 | 0.038 | 0.069 | 0 | 0.042 | 0 | 0.132 | 0.091 | 0.029 | 0.295 | 0.083 | 0 | 0.114 | 0.02 | 0.038 |
| H_11 | 0 | 0 | **0.029** | 0 | 0 | 0 | 0 | 0 | 0 | 0 | 0 | 0 | 0 | 0 | 0 | 0 | 0 |
| H_12 | 0 | 0 | **0.059** | 0 | 0 | 0 | 0 | 0 | 0 | 0 | 0 | 0 | 0 | 0 | 0 | 0 | 0 |
| H_13* | 0 | 0 | 0.029 | 0 | 0 | 0 | 0 | 0 | 0 | 0 | 0 | 0 | 0.021 | 0 | 0 | 0 | 0 |
| H_14 | 0 | 0 | 0 | 0.077 | 0 | 0 | 0 | 0 | 0 | 0 | 0 | 0 | 0 | 0 | 0 | 0 | 0.038 |
| H_15 | 0 | 0 | 0 | **0.038** | 0 | 0 | 0 | 0 | 0 | 0 | 0 | 0 | 0 | 0 | 0 | 0 | 0 |
| H_16 | 0 | 0 | 0 | 0.038 | 0 | 0 | 0 | 0 | 0 | 0 | 0 | 0 | 0.021 | 0 | 0 | 0 | 0 |
| H_17 | 0 | 0 | 0 | **0.038** | 0 | 0 | 0 | 0 | 0 | 0 | 0 | 0 | 0 | 0 | 0 | 0 | 0 |
| H_18 | 0 | 0 | 0 | 0 | 0.034 | 0 | 0 | 0.056 | 0 | 0 | 0 | 0 | 0 | 0 | 0 | 0 | 0 |
| H_19 | 0 | 0 | 0 | 0 | **0.017** | 0 | 0 | 0 | 0 | 0 | 0 | 0 | 0 | 0 | 0 | 0 | 0 |
| H_20 | 0 | 0 | 0 | 0 | 0.121 | 0.079 | 0.167 | 0.111 | 0.026 | 0.136 | 0.059 | 0 | 0.063 | 0.25 | 0.091 | 0.143 | 0 |
| H_21 | 0 | 0 | 0 | 0 | 0.017 | 0.053 | 0.042 | 0.444 | 0 | 0 | 0 | 0 | 0 | 0 | 0 | 0 | 0 |
| H_22 | 0 | 0 | 0 | 0 | 0.017 | 0 | 0 | 0 | 0 | 0 | 0.059 | 0 | 0.042 | 0.031 | 0 | 0 | 0 |
| H_23 | 0 | 0 | 0 | 0 | 0 | 0.053 | 0.042 | 0 | 0 | 0 | 0 | 0.045 | 0 | 0 | 0 | 0 | 0 |
| H_24 | 0 | 0 | 0 | 0 | 0 | **0.026** | 0 | 0 | 0 | 0 | 0 | 0 | 0 | 0 | 0 | 0 | 0 |
| H_25 | 0 | 0 | 0 | 0 | 0 | **0.026** | 0 | 0 | 0 | 0 | 0 | 0 | 0 | 0 | 0 | 0 | 0 |
| H_26 | 0 | 0 | 0 | 0 | 0 | **0.026** | 0 | 0 | 0 | 0 | 0 | 0 | 0 | 0 | 0 | 0 | 0 |
| H_27 | 0 | 0 | 0 | 0 | 0 | **0.053** | 0 | 0 | 0 | 0 | 0 | 0 | 0 | 0 | 0 | 0 | 0 |
| H_28 | 0 | 0 | 0 | 0 | 0 | 0.026 | 0.042 | 0 | 0 | 0 | 0 | 0 | 0 | 0 | 0 | 0 | 0 |
| H_29 | 0 | 0 | 0 | 0 | 0 | 0.026 | 0.042 | 0 | 0 | 0 | 0 | 0 | 0.042 | 0 | 0 | 0.02 | 0 |
| H_30 | 0 | 0 | 0 | 0 | 0 | 0 | **0.042** | 0 | 0 | 0 | 0 | 0 | 0 | 0 | 0 | 0 | 0 |
| H_31 | 0 | 0 | 0 | 0 | 0 | 0 | 0.042 | 0 | 0.026 | 0 | 0 | 0 | 0 | 0.031 | 0 | 0 | 0 |
| H_32 | 0 | 0 | 0 | 0 | 0 | 0 | 0.042 | 0 | 0.026 | 0 | 0 | 0 | 0 | 0 | 0.023 | 0 | 0 |
| H_33 | 0 | 0 | 0 | 0 | 0 | 0 | 0.042 | 0 | 0 | 0 | 0 | 0 | 0.021 | 0 | 0 | 0 | 0 |
| H_34 | 0 | 0 | 0 | 0 | 0 | 0 | **0.042** | 0 | 0 | 0 | 0 | 0 | 0 | 0 | 0 | 0 | 0 |
| H_35 | 0 | 0 | 0 | 0 | 0 | 0 | 0.042 | 0 | 0 | 0 | 0 | 0 | 0 | 0 | 0.023 | 0 | 0 |
| H_36 | 0 | 0 | 0 | 0 | 0 | 0 | 0.042 | 0 | 0 | 0 | 0.029 | 0 | 0 | 0 | 0 | 0 | 0 |
| H_37 | 0 | 0 | 0 | 0 | 0 | 0 | **0.042** | 0 | 0 | 0 | 0 | 0 | 0 | 0 | 0 | 0 | 0 |
| H_38 | 0 | 0 | 0 | 0 | 0 | 0 | 0 | **0.028** | 0 | 0 | 0 | 0 | 0 | 0 | 0 | 0 | 0 |
| H_39* | 0 | 0 | 0 | 0 | 0 | 0 | 0 | **0.028** | 0 | 0 | 0 | 0 | 0 | 0 | 0 | 0 | 0 |
| H_40 | 0 | 0 | 0 | 0 | 0 | 0 | 0 | **0.028** | 0 | 0 | 0 | 0 | 0 | 0 | 0 | 0 | 0 |
| H_41 | 0 | 0 | 0 | 0 | 0 | 0 | 0 | **0.056** | 0 | 0 | 0 | 0 | 0 | 0 | 0 | 0 | 0 |
| H_42 | 0 | 0 | 0 | 0 | 0 | 0 | 0 | **0.028** | 0 | 0 | 0 | 0 | 0 | 0 | 0 | 0 | 0 |
| H_43* | 0 | 0 | 0 | 0 | 0 | 0 | 0 | **0.056** | 0 | 0 | 0 | 0 | 0 | 0 | 0 | 0 | 0 |
| H_44 | 0 | 0 | 0 | 0 | 0 | 0 | 0 | **0.056** | 0 | 0 | 0 | 0 | 0 | 0 | 0 | 0 | 0 |
| H_45 | 0 | 0 | 0 | 0 | 0 | 0 | 0 | **0.028** | 0 | 0 | 0 | 0 | 0 | 0 | 0 | 0 | 0 |
| H_46 | 0 | 0 | 0 | 0 | 0 | 0 | 0 | 0 | **0.026** | 0 | 0 | 0 | 0 | 0 | 0 | 0 | 0 |
| H_47 | 0 | 0 | 0 | 0 | 0 | 0 | 0 | 0 | 0.026 | 0 | 0 | 0 | 0.021 | 0 | 0 | 0 | 0 |
| H_48 | 0 | 0 | 0 | 0 | 0 | 0 | 0 | 0 | 0 | 0.091 | 0.059 | 0 | 0 | 0 | 0 | 0.041 | 0 |
| H_49 | 0 | 0 | 0 | 0 | 0 | 0 | 0 | 0 | 0 | **0.091** | 0 | 0 | 0 | 0 | 0 | 0 | 0 |
| H_50* | 0 | 0 | 0 | 0 | 0 | 0 | 0 | 0 | 0 | 0 | **0.029** | 0 | 0 | 0 | 0 | 0 | 0 |
| H_51 | 0 | 0 | 0 | 0 | 0 | 0 | 0 | 0 | 0 | 0 | **0.029** | 0 | 0 | 0 | 0 | 0 | 0 |
| H_52 | 0 | 0 | 0 | 0 | 0 | 0 | 0 | 0 | 0 | 0 | 0 | 0 | **0.021** | 0 | 0 | 0 | 0 |
| H_53 | 0 | 0 | 0 | 0 | 0 | 0 | 0 | 0 | 0 | 0 | 0 | 0 | **0.021** | 0 | 0 | 0 | 0 |
| H_54 | 0 | 0 | 0 | 0 | 0 | 0 | 0 | 0 | 0 | 0 | 0 | 0 | **0.021** | 0 | 0 | 0 | 0 |
| H_55 | 0 | 0 | 0 | 0 | 0 | 0 | 0 | 0 | 0 | 0 | 0 | 0 | 0 | 0 | **0.023** | 0 | 0 |
| H_56 | 0 | 0 | 0 | 0 | 0 | 0 | 0 | 0 | 0 | 0 | 0 | 0 | 0 | 0 | 0 | 0 | **0.23** |
| H_57 | 0 | 0 | 0 | 0 | 0 | 0 | 0 | 0 | 0 | 0 | 0 | 0 | 0 | 0 | 0 | 0 | **0.04** |
| H_58 | 0 | 0 | 0 | 0 | 0 | 0 | 0 | 0 | 0 | 0 | 0 | 0 | 0 | 0 | 0 | 0 | **0.04** |
| H_59 | 0 | 0 | 0 | 0 | 0 | 0 | 0 | 0 | 0 | 0 | 0 | 0 | 0 | 0 | 0 | 0 | **0.04** |
| H_60 | 0 | 0 | 0 | 0 | 0 | 0 | 0 | 0 | 0 | 0 | 0 | 0 | 0 | 0 | 0 | 0 | **0.04** |
| H_61 | 0 | 0 | 0 | 0 | 0 | 0 | 0 | 0 | 0 | 0 | 0 | 0 | 0 | 0 | 0 | **0.02** | 0 |
