# Supplementary material for: The Whereabouts of an Ancient Wanderer: Global Phylogeography of the Solitary Ascidian Styela plicata
Source: PLoS One. 2011 Sep 23;6(9):e25495. doi: 10.1371/journal.pone.0025495 (PMC3179514; doi:10.1371/journal.pone.0025495)
Supplement: Table S3 — ANT allelic phase and COI haplotypes for each individual analyzed. (DOC) [file pone.0025495.s003.doc]

| Ind. | *ANT* | *COI* |  | Ind. | *ANT* | *COI* |  | Ind. | *ANT* | *COI* |  | Ind. | *ANT* | *COI* |
| --- | --- | --- | --- | --- | --- | --- | --- | --- | --- | --- | --- | --- | --- | --- |
| AR1 | H1,H2 | H1 |  | TEN13 | H2,H8 | H2 |  | SC11 | H10,H46 | H8 |  | OKI3 | H2,H20 | H19 |
| AR2 | H2,H2 | H1 |  | TEN14 | H8,H21 | H2 |  | SC12 | H1,H2 | H8 |  | OKI4 | H2,H8 | H19 |
| AR3 | H2,H2 | H1 |  | TEN15 | H2,H8 | H2 |  | SC13 | H2,H31 | H17 |  | OKI5 | - | H19 |
| AR4 | H2,H2 | H1 |  | TEN16 | H8,H20 | H2 |  | SC14 | H1,H5 | H2 |  | OKI6 | H2,H8 | H19 |
| AR5 | H2,H3 | H1 |  | TEN17 | H8,H8 | H2 |  | SC15 | H2,H8 | H8 |  | OKI7 | - | H2 |
| AR6 | H4,H5 | H1 |  | TEN19 | H1,H22 | - |  | SC16 | H8,H10 | H8 |  | OKI8 | H2,H20 | H19 |
| AR7 | H2,H2 | H1 |  | TEN20 | H8,H8 | - |  | SC17 | H1H2 | H2 |  | OKI9 | H2,H20 | H19 |
| AR8 | H1,H6 | H1 |  | TEN21 | H8,H10 | H2 |  | SC18 | H1,H20 | H17 |  | OKI10 | H6,H8 | H19 |
| AR9 | - | H1 |  | TEN22 | H2,H20 | H2 |  | SC19 | H4,H8 | H2 |  | OKI11 | - | H19 |
| AR10 | H2,H2 | H1 |  | TEN23 | H2,H8 | H2 |  | SC20 | H1,H8 | H8 |  | OKI12 | H2,H8 | H19 |
| AR11 | H2,H4 | H1 |  | TEN24 | H3,H20 | H2 |  | SC21 | - | H2 |  | OKI13 | - | H19 |
| AR12 | H2,H2 | H1 |  | TEN25 | H8,H10 | H2 |  | SC22 | H2,H10 | H18 |  | OKI14 | H2,H31 | H19 |
| AR13 | H1,H1 | H1 |  | TEN26 | H2,H8 | - |  | SC23 | - | H8 |  | OKI15 | H2,H8 | H2 |
| AR14 | H4,H4 | H1 |  | TEN27 | H10,H20 | - |  | SC24 | H10,H47 | H19 |  | OKI16 | H20,H20 | H1 |
| AR15 | H1,H2 | H1 |  | TEN28 | H8,H10 | H2 |  | SC25 | - | H19 |  | OKI17 | - | H19 |
| AR16 | H2,H4 | H2 |  | TEN29 | H2,H8 | H2 |  | CAL1 | H2,H48 | H1 |  | OKI18 | H2,H8 | H1 |
| AR17 | H1,H4 | H1 |  | TEN30 | H2,H20 | - |  | CAL2 | H8,H49 | H20 |  | OKI19 | H1,H22 | H1 |
| AR18 | H2,H4 | H1 |  | TEN31 | H2,H8 | H2 |  | CAL3 | H2,H20 | H5 |  | OKI20 | - | H19 |
| AR19 | H2,H2 | H1 |  | KNY1 | H23,H23 | H2 |  | CAL4 | H2,H20 | H20 |  | OKI21 | H2,H8 | H19 |
| AR20 | H2,H2 | H1 |  | KNY2 | H2,H2 | H2 |  | CAL5 | H2,H20 | H5 |  | OKI22 | H2,H20 | H19 |
| JA1 | H4,H4 | H3 |  | KNY3 | - | H2 |  | CAL6 | H2,H8 | H5 |  | OKI23 | - | H2 |
| JA2 | H2,H2 | H1 |  | KNY4 | H6,H8 | - |  | CAL7 | H8,H10 | H1 |  | OKI24 | - | H19 |
| JA3 | H2,H2 | H1 |  | KNY5 | H2,H8 | H3 |  | CAL8 | H2,H8 | H2 |  | OKI25 | - | H19 |
| JA4 | H1,H1 | H4 |  | KNY6 | - | H1 |  | CAL9 | H2,H48 | H5 |  | MIS1 | H2,H8 | H5 |
| JA5 | H2,H7 | - |  | KNY7 | H2,H8 | H1 |  | CAL10 | H8,H10 | H1 |  | MIS2 | H2,H8 | H5 |
| JA6 | H2,H2 | - |  | KNY8 | - | H1 |  | CAL11 | H8,H49 | H2 |  | MIS3 | H8,H10 | H2 |
| JA7 | H2,H2 | H1 |  | KNY9 | H2,H24 | H1 |  | BRA1 | H2,H8 | - |  | MIS4 | H20,H20 | H5 |
| JA8 | - | H1 |  | KNY10 | - | H2 |  | BRA3 | H2,H20 | - |  | MIS5 | - | H5 |
| JA10 | H2,H2 | H3 |  | KNY11 | H2,H25 | - |  | BRA5 | - | H2 |  | MIS6 | H1,H8 | H5 |
| JA11 | H6,H6 | H1 |  | KNY12 | H2,H26 | H1 |  | BRA7 | H2,H8 | H5 |  | MIS7 | - | H5 |
| JA12 | H2,H2 | H1 |  | KNY13 | H20,H20 | - |  | BRA8 | H2,H8 | H5 |  | MIS8 | H10,H35 | H5 |
| JA13 | - | H1 |  | KNY14 | H2,H8 | H1 |  | BRA9 | H2,H8 | H2 |  | MIS9 | H8,H10 | H5 |
| JA14 | H2,H2 | H4 |  | KNY15 | H6,H27 | H2 |  | BRA10 | - | H5 |  | MIS10 | H2,H8 | H5 |
| JA15 | H2,H2 | H1 |  | KNY16 | - | H3 |  | BRA11 | H6,H8 | H5 |  | MIS11 | H2,H20 | H5 |
| JA16 | H2,H2 | H1 |  | KNY17 | H2,H27 | H2 |  | BRA12 | H2,H22 | H2 |  | MIS12 | H8,H55 | H5 |
| JA17 | H1,H1 | H3 |  | KNY18 | - | H3 |  | BRA13 | H2,H48 | H2 |  | MIS13 | H5,H8 | H5 |
| JA18 | H2,H2 | H1 |  | KNY19 | H2,H8 | H2 |  | BRA16 | H8,H50 | H2 |  | MIS14 | H2,H8 | H5 |
| JA19 | H2,H2 | H1 |  | KNY20 | H8,H28 | H1 |  | BRA17 | - | H2 |  | MIS15 | H2,H8 | H5 |
| JA20 | H2,H2 | H3 |  | KNY21 | H2,H20 | H10 |  | BRA18 | H36,H51 | H2 |  | MIS16 | H2,H8 | H5 |
| JA22 | H2,H4 | H1 |  | KNY22 | H2,H8 | H2 |  | BRA19 | H2,H8 | H2 |  | MIS17 | H8,H10 | H5 |
| JA23 | H2,H2 | H1 |  | KNY23 | H21,H29 | H1 |  | BRA20 | H8,H10 | H2 |  | MIS18 | H2,H8 | H5 |
| JA24 | H4,H4 | H1 |  | KNY24 | H2,H8 | H2 |  | BRA21 | H2,H20 | H2 |  | MIS19 | H8,H10 | H5 |
| SP2 | H8,H9 | H5 |  | KNY25 | H8,H21 | H1 |  | BRA22 | H2,H22 | H8 |  | MIS20 | H2,H8 | H19 |
| SP4 | H2,H2 | H6 |  | PE2 | H2,H20 | H5 |  | BRA23 | H2,H8 | H2 |  | MIS21 | H2,H20 | H19 |
| SP5 | H1,H10 | H1 |  | PE3 | - | H5 |  | BRA24 | - | H2 |  | MIS22 | H4,H8 | H19 |
| SP6 | H4,H10 | H2 |  | PE4 | - | H1 |  | BRA25 | H2,H8 | H2 |  | MIS23 | H1,H8 | H5 |
| SP9 | H2,H8 | H5 |  | PE5 | H1,H30 | - |  | BRA26 | H2,H48 | H8 |  | MIS24 | H2,H32 | H5 |
| SP10 | - | H1 |  | PE6 | - | H5 |  | AM1 | H2,H10 | H5 |  | MIS25 | - | H19 |
| SP11 | H2,H2 | H5 |  | PE7 | H28,H31 | H5 |  | AM2 | H2,H10 | H10 |  | SKS1 | H2,H8 | H1 |
| SP12 | H6,H6 | - |  | PE8 | - | H2 |  | AM3 | H2,H10 | H5 |  | SKS2 | H2,H8 | H2 |
| SP14 | H2,H2 | H5 |  | PE9 | H10,H32 | H5 |  | AM4 | H10,H10 | H5 |  | SKS3 | H2,H48 | H2 |
| SP15 | H2,H11 | H7 |  | PE10 | - | H5 |  | AM5 | H1,H1 | H5 |  | SKS4 | H2,H8 | H2 |
| SP16 | H1,H9 | - |  | PE11 | - | H5 |  | AM6 | H2,H2 | H5 |  | SKS5 | H2,H8 | H1 |
| SP18 | H1,H2 | H2 |  | PE13 | H21,H33 | - |  | AM9 | H2,H10 | H5 |  | SKS6 | H2,H8 | H1 |
| SP20 | H2,H12 | H2 |  | PE14 | - | H5 |  | AM10 | H2,H2 | H5 |  | SKS7 | H2,H8 | H2 |
| SP21 | H2,H12 | H7 |  | PE15 | H8,H23 | H5 |  | AM11 | H2,H2 | H5 |  | SKS8 | H2,H61 | H19 |
| SP22 | H4,H13 | H2 |  | PE16 | H2,H34 | H5 |  | AM12 | H2,H2 | H5 |  | SKS9 | H2,H8 | H2 |
| SP23 | - | H2 |  | PE17 | H20,H20 | H5 |  | AM13 | H2,H10 | H5 |  | SKS10 | H2,H8 | H1 |
| SP24 | H2,H2 | H2 |  | PE18 | H2,H20 | H5 |  | AM14 | H2,H10 | H5 |  | SKS11 | H8,H20 | H21 |
| SP26 | H2,H2 | H6 |  | PE19 | - | H5 |  | AM15 | H1,H5 | H5 |  | SKS12 | H2,H8 | H2 |
| SP29 | H5,H5 | - |  | PE20 | - | H5 |  | AM16 | H2,H10 | H5 |  | SKS13 | H2,H20 | H19 |
| FE1 | H2,H2 | - |  | PE21 | H2,H35 | - |  | AM17 | H23,H23 | H5 |  | SKS14 | H2,H8 | H1 |
| FE2 | H2,H2 | H2 |  | PE22 | H29,H36 | H5 |  | AM18 | H2,H2 | H5 |  | SKS15 | H2,H20 | H19 |
| FE3 | H1,H2 | H8 |  | PE23 | H8,H37 | H5 |  | AM19 | H2,H2 | H5 |  | SKS16 | H2,H8 | H1 |
| FE4 | H2,H2 | H9 |  | PE24 | - | H5 |  | AM20 | H2,H10 | H5 |  | SKS17 | H2,H8 | H1 |
| FE5 | H4,H4 | H10 |  | NC1 | H21,H21 | H11 |  | AM21 | - | H5 |  | SKS18 | H1,H48 | H2 |
| FE6 | H14,H14 | H1 |  | NC2 | H3,H3 | H12 |  | AM22 | H2,H2 | H5 |  | SKS19 | H2,H8 | H1 |
| FE7 | - | H8 |  | NC4 | H21,H38 | H11 |  | AM23 | H2,H10 | H5 |  | SKS20 | H2,H20 | H19 |
| FE8 | H1,H15 | H5 |  | NC5 | H4,H21 | H11 |  | AM26 | H10,H10 | H5 |  | SKS21 | H2,H20 | H1 |
| FE9 | - | H1 |  | NC6 | H21,H21 | H11 |  | AM29 | - | H5 |  | SKS22 | H2,H20 | H1 |
| FE10 | - | H2 |  | NC7 | - | H12 |  | AM30 | H2,H2 | H5 |  | SKS23 | H10,H20 | H1 |
| FE11 | - | H1 |  | NC8 | - | H12 |  | WAK1 | H8,H10 | H1 |  | SKS24 | H2,H29 | - |
| FE12 | H3,H4 | H8 |  | NC9 | H21,H21 | H11 |  | WAK2 | H5,H8 | H2 |  | SKS25 | H2,H2 | H1 |
| FE14 | - | H5 |  | NC10 | H21,H21 | H13 |  | WAK3 | H2,H8 | H2 |  | HK1 | H14,H56 | H1 |
| FE15 | - | H2 |  | NC11 | H39,H40 | H11 |  | WAK4 | H8,H10 | H19 |  | HK2 | H2,H10 | H1 |
| FE16 | - | H2 |  | NC12 | H41,H41 | H13 |  | WAK5 | H2,H33 | H2 |  | HK3 | H2,H57 | H10 |
| FE17 | H4,H16 | H2 |  | NC13 | H18,H18 | H11 |  | WAK6 | H8,H10 | H2 |  | HK4 | H4,H58 | H1 |
| FE18 | H2,H3 | - |  | NC14 | H20,H20 | H11 |  | WAK7 | H2,H8 | H2 |  | HK5 | - | H10 |
| FE19 | - | H1 |  | NC15 | H21,H42 | H13 |  | WAK8 | H8,H10 | H2 |  | HK6 | H3,H5 | H1 |
| FE20 | H3,H17 | H2 |  | NC16 | - | H14 |  | WAK9 | H2,H47 | H19 |  | HK7 | H59,H60 | H2 |
| FE21 | - | H1 |  | NC17 | H43,H43 | H15 |  | WAK10 | H2,H22 | H2 |  | HK9 | - | H1 |
| FE22 | - | H2 |  | NC18 | H20,H20 | H12 |  | WAK11 | H8,H8 | H2 |  | HK10 | - | H2 |
| FE23 | - | H1 |  | NC19 | H21,H21 | H11 |  | WAK12 | H2,H8 | H19 |  | HK11 | - | H2 |
| FE24 | - | H8 |  | NC20 | H44,H45 | H11 |  | WAK13 | H2,H22 | H2 |  | HK12 | - | H19 |
| FE25 | H1,H2 | - |  | NC21 | - | H11 |  | WAK14 | H2,H13 | H2 |  | HK13 | - | H2 |
| FE26 | H2,H10 | - |  | NC22 | H21,H44 | H11 |  | WAK15 | H8,H8 | H19 |  | HK14 | - | H2 |
| TEN1 | H2,H8 | H2 |  | NC23 | - | H13 |  | WAK16 | H8,H52 | H2 |  | HK15 | - | H1 |
| TEN2 | H2,H8 | H2 |  | NC25 | H21,H21 | H16 |  | WAK17 | H16,H53 | H2 |  | HK16 | - | H2 |
| TEN3 | H8,H18 | H2 |  | SC1 | H2,H10 | H8 |  | WAK18 | - | H2 |  | HK17 | H4,H60 | H2 |
| TEN4 | H2,H8 | H2 |  | SC2 | - | H8 |  | WAK19 | H2,H29 | H1 |  | HK18 | H4,H56 | H1 |
| TEN5 | H2,H8 | H2 |  | SC3 | H4,H8 | H2 |  | WAK20 | H8,H54 | H19 |  | HK19 | H2,H4 | H2 |
| TEN6 | H2,H19 | H2 |  | SC4 | H2,H2 | H2 |  | WAK21 | H20,H20 | H19 |  | HK20 | H4,H56 | H2 |
| TEN7 | H2,H8 | - |  | SC5 | H2,H32 | H2 |  | WAK22 | H8,H8 | H2 |  | HK21 | H4,H56 | - |
| TEN8 | H8,H18 | H2 |  | SC6 | - | H2 |  | WAK23 | H2,H29 | H19 |  | HK22 | - | H22 |
| TEN9 | H2,H8 | H2 |  | SC7 | H2,H2 | H8 |  | WAK24 | H8,H20 | H2 |  | HK23 | H4,H56 | H1 |
| TEN10 | - | H2 |  | SC8 | - | H8 |  | WAK25 | H2,H8 | H2 |  | HK24 | - | H2 |
| TEN11 | H2,H20 | H2 |  | SC9 | H2,H2 | H17 |  | OKI1 | H1,H20 | H19 |  | HK25 | - | H19 |
| TEN12 | H2,H8 | H2 |  | SC10 | H2,H2 | H8 |  | OKI2 | H2,H20 | - |  | HK27 | H4,H56 | H2 |
